# Efficacy of Metacognitive Training for Patients With Schizophrenia in Psychiatric Emergency Wards: A Pilot Randomized Controlled Trial

**DOI:** 10.3389/fpsyg.2022.861102

**Published:** 2022-04-11

**Authors:** Saori Haga, Masayoshi Kobayashi, Ayako Takehara, Kojiro Kawano, Kenji Endo

**Affiliations:** ^1^Department of Rehabilitation, Tikumaso Mental Hospital, Ueda, Japan; ^2^Department of Health Sciences, Graduate School of Medicine, Shinshu University, Matsumoto, Japan

**Keywords:** metacognitive training (MCT), schizophrenia, emergency psychiatric ward, occupational therapy, cognitive bias

## Abstract

**Introduction:**

Metacognitive training (MCT) is a group program for improving cognitive bias in patients with schizophrenia. MCT has a reported positive effect on psychiatric symptoms and cognitive bias in patients with schizophrenia, but the effect of the intervention on patients with schizophrenia in the early recovery stage during hospitalization is not comprehensible. Therefore, this study aimed to investigate the efficacy of MCT in the early recovery stage of patients with schizophrenia in a Japanese emergency psychiatric ward.

**Method:**

This unblinded, pilot randomized controlled trial recruited 24 patients with schizophrenia aged 20–65 years. Patients were randomly divided into two groups: occupational therapy (OT) + MCT group and OT-only group. Using the two-way repeated-measures analysis of variance (ANOVA), changes in cognitive function, psychiatric symptoms, cognitive insight, and intrinsic motivation were compared between those at baseline and post-intervention and between the two groups. Furthermore, patient readmission during the year after discharge was compared between the groups.

**Results:**

The final analysis included eight patients in each group, owing to the withdrawal of some patients from the study. The two-way repeated-measures analysis of variance revealed significant differences in cognitive function in several domains within subjects. However, no significant differences between subjects were observed. Psychiatric symptoms showed significant within-subject improvement, and interaction was found for general psychopathology (*p* = 0.03). The variable of cognitive insight and self-reflectiveness was significantly different between subjects (*p* = 0.03). There was no significant difference in intrinsic motivation. Readmission within a year was significantly lower in the OT + MCT group than in the OT-only group (2 [25%] vs. 6 [75%]; *p* = 0.046).

**Conclusion:**

In a Japanese emergency psychiatric ward, this pilot randomized controlled study was the first attempt to investigate the efficacy of MCT in patients with schizophrenia suggesting that MCT may be effective in preventing psychiatric symptoms, poor self-reflectiveness, and readmissions.

The study was registered in the University Hospital Medical Information Network Clinical Trials Registry (UMIN-CTR; UMIN000034106).

## Introduction

As reported in the quality of mental health care in Japan (OECD, 2015), Japanese mental health care faces challenges such as high suicide rates, high numbers of mental health care beds, and long lengths of stay. These issues have revealed a slight downward trend recently; however, the number of psychiatric beds per 1,000 people is 2.7, and the average length of stay (268 days) are still high (Ministry of Health, Labor and Welfare, 2018). A psychiatric emergency ward has been institutionalized to allow more than 60% of inpatients to be discharged within 3 months; however, no treatment program was stipulated. The Japanese Association of Emergency Psychiatry (2015) states that psychosocial treatment strategies for inpatients include psychoeducation, social skills training (SST), and occupational therapy for convalescent patients. However, no specific intervention strategy has been defined. For this reason, it is important to clarify specific psychosocial treatment strategies for psychiatric inpatients.

In recent years, cognitive dysfunction has been considered the core pathology of schizophrenia (Green, 1996; Green et al., 2000), and attention has been given to rehabilitation to improve cognitive function. Schizophrenic cognitive dysfunction involves neurocognitive deficits in attention, verbal memory, working memory, verbal fluency, executive function, processing speed; as well as a tendency for cognitive bias to occur readily in areas such as the theory of mind (e.g., being able to see things from someone else’s point of view), causal-attribution bias (e.g., searching for external factors as causes of undesirable events), and jumping to conclusions (e.g., hastiness in reaching conclusions, making judgments with limited information and excessive degree of certainty). These cognitive biases are associated with delusions and misplaced certainty, resulting in negative effects on the patients’ social functions, such as their daily living, interpersonal relations, and work (Nicolò et al., 2012). The efficacy of drug therapy for treating cognitive dysfunction is limited (Keefe and Harvey, 2012); therefore, it is necessary to provide therapeutic intervention to alleviate neurocognitive and social cognitive disorders for patients with schizophrenia who are hospitalized in the psychiatric emergency ward.

Moritz and Woodward (2007) and Moritz et al. (2011, 2014a,2014b) developed metacognitive training (MCT) to alleviate cognitive bias in schizophrenia. MCT is based on the fundamental principles of cognitive-behavioral therapy and is positioned as a psychoeducation program implemented on a group or individual basis. In MCT sessions, modules (PowerPoint materials) prepared by learning-related themes are presented to the participants; and employing group discussions, the participants repeatedly practice recognizing their cognitive biases. The aim is then to help individual participants recognize their own thinking biases and then come up with alternative ways of thinking to promote more appropriate cognition. Previous studies have shown that MCT reduces positive symptoms in patients with schizophrenia (Moritz and Woodward, 2007; Kumar et al., 2010; Moritz et al., 2011, 2014a; Briki et al., 2014; Pankowski et al., 2016). In addition, it has been reported that improvements are maintained for as long as three years (Moritz et al., 2014b).

The Japanese version of the MCT was developed in 2012 (Ishigaki, 2012). Several attempts were made to verify the efficacy of MCT in Japanese mental health services, but few reports have been published (Hosono et al., 2013; Morimoto et al., 2015). The first randomized, comparative Japanese study was published recently (Ishikawa et al., 2020), which was a multi-site study assessing the efficacy of the Japanese version of the MCT in patients with schizophrenia. It was shown that MCT-alleviated positive symptoms, especially delusional symptoms, improved patients’ general functioning and alleviated the cognitive bias. Furthermore, a pilot study in long-term hospitalized schizophrenic patients suggests that MCT may improve patients’ neurocognitive function, particularly verbal memory and attention (Fujii et al., 2021).

The present study investigated whether the positive symptoms and cognitive bias of patients with schizophrenia were alleviated by MCT while improving intrinsic motivation and neurocognitive functions such as attention and verbal memory at the same time or in connection with this alleviation. In addition, the aim is to attract participants’ attention by employing interesting and interactive MCT sessions^1^; this approach might be useful for patients in the early recovery stage of schizophrenia even immediately after admission to a mental hospital. However, the effects of MCT on neurocognitive function and intrinsic motivation have not been investigated to date, and there have been no reports of the use of MCT for schizophrenia patients admitted to emergency psychiatric or acute-care wards. In the present randomized comparative study, MCT was introduced to schizophrenia patients who were undergoing occupational therapy (OT) while hospitalized in Japanese emergency psychiatric wards to investigate its efficacy. If the efficacy of MCT can be demonstrated, this will provide a rationale for including MCT in OT programs in emergency psychiatric wards, which should lead to significant progress in schizophrenic rehabilitation.

## Materials and Methods

### Participants

The study was performed with patients who: (a) were hospitalized in the emergency ward of Tikumaso Mental Hospital between 3 October 2018 and 31 October 2020; (b) were diagnosed by the attending physician, by the diagnostic criteria in the Fifth Edition of the Diagnostic and Statistical Manual (DSM-5) (American Psychiatric Association [APA], 2013), as having schizophrenia or schizoaffective disorder; (c) were treated by OT; and (d) met none of the following exclusion criteria: (i) aged under 20 or over 65; (ii) with one or more of the following complications: intellectual disability, alcohol or drug abuse or dependency, dementia, epilepsy, cranial trauma, or cerebrovascular disease; (iii) in an unstable condition, making it difficult to perform MCT and/or cognitive function tests; (iv) refused to participate in the study; and (v) determined to be inappropriate for participation by the attending physician or investigator. All prospective patients were given written and oral explanations of the study, and informed consent to participate was obtained from each participant.

### Research Ethics

This study was approved by the Medical Corporation Yuaikai Tikumaso Mental Hospital Ethics Review Committee (approval number: 1873, 2018) and the Shinshu University School of Medicine Medical Ethics Committee (approval number: 4151, 2018).

### Study Design and Procedures

The study design was randomized, comparative, unblinded, and patients who gave informed consent to participate were allocated to the OT + MCT or OT-only group by a stratified randomization method based on age and sex. The allocation-adjustment factors for stratified block randomization were (i) age: 20–30, 31–40, 41–50, and 51–64 years; (ii) sex: male and female. Baseline (i.e., pre-intervention) evaluation was performed after the allocation. In the OT + MCT group, MCT was implemented twice per week, alongside the usual OT program, making a total of 16 MCT sessions during a hospitalization period of 2–3 months. In the OT-only group, the usual OT program was implemented. The post-intervention evaluation was performed after the completion of MCT or at discharge from the hospital. In the OT + MCT group, patients who participated in eight or more of the 16 MCT sessions (≥50%) were included in the analyses.

The Japanese version of the MCT (Ishigaki, 2012), which consists of eight modules, was used. For this MCT program, two series were prepared, each with eight modules covering causal attribution, jumping to conclusions, confirmation bias, theory of mind, errors in verbal memory, and self-esteem. In this study, two MCT sessions, with two themes, were held each week, each session lasting 45–60 min. The sessions were held in semi-closed groups, each containing three to four patients, and irrespective of the theme at any time, new participants were accepted. All MCT sessions were managed by the same occupational therapist, who directed the program following the methods stipulated in the MCT Manual (Moritz et al., 2010). In each MCT session, the occupational therapist presented PowerPoint materials for each module, using a projector, encouraged the patients to talk to each other about the issues raised, and tried to get them to tackle the issues to be learned, at the same time as enjoying themselves. The usual OT, in contrast, was an individual or group program, with four to five sessions per week, each lasting 1–2 h, per the patients’ wishes and rehabilitation targets; including light exercise, craftwork, recreation, psychoeducation, and support in preparation for discharge from hospital.

In addition, readmission within 1 year after discharge was investigated, and a comparison of the readmission rate between the OT + MCT group and the OT alone group was made.

### Measurements

The demographic information of the participants, including age, gender, cohabitation, education period, disease duration, number of hospital admissions, the period until the start of the OT, OT implementation timing, hospitalization period, and antipsychotic dose, were investigated.

The Japanese version of the Brief Assessment of Cognition in Schizophrenia (BACS) (Keefe et al., 2004; Kaneda et al., 2007, 2013) was used to evaluate cognitive function. The BACS assesses multiple aspects of cognitive functioning in schizophrenia and includes six measures: verbal memory, working memory, motor speed, verbal fluency, attention, and executive functioning. Each of the six measures was standardized by z-scores, whereby the mean scores of the healthy participants were set to zero, and the standard deviations were set to one. The composite score was calculated by averaging all the z-scores of the six BACS measures. As a yardstick for severity, −0.5 ≦−1 was considered a mild disorder, −1.0 ≦−1.5 was considered to be a moderate disorder, and ≤−1.5 was considered to be a severe disorder.

The symptoms were assessed using the Positive and Negative Syndrome Scale (PANSS) (Kay et al., 1987). The PANSS is a 30-item rating scale designed to assess the severity of psychotic symptoms. PANSS outcomes were analyzed using a 3-factor solution which included positive, negative, and general psychopathology. All items were rated 1 (*absent*) to 7 (*extreme*), with higher scores indicating more severe symptoms.

The Beck Cognitive Insight Scale (BCIS) was used to assess cognitive insights. The BCIS, created by Beck et al. (2004), is a self-report evaluation scale that measures cognitive bias. The respondents were asked to rate how much they agreed with each of the 15 items using a 4-point scale (0 = do not agree at all, 3 = completely agree). Self-Reflectiveness (SR) was compiled from nine items (range: 0–27) and self-certainty (SC) from six items (range: 0–18). The higher the SR score, the higher-level insight a person has toward their thoughts, and the higher the SC score, the stronger a person is convinced of their thinking. A composite index is calculated by subtracting the SC score from the SR score (range: −18 to 27), and the higher the score, the more appropriate a person’s cognitive tendencies are judged to be. In this study, the Japanese language edition of the BCIS produced by Uchida et al. (2009) was used and evaluated by occupational therapists. The Japanese version of the BCIS has been reported to be effective, reliable, and tolerable (Cronbach’s α = 0.67–0.78).

Intrinsic motivation was assessed using the Japanese version of the Intrinsic Motivation Inventory (IMI) (Choi et al., 2010). The IMI is a 21-item self-report scale that measures interest/enjoyment, perceived choice, and value/usefulness. Items were answered on a 7-point Likert scale with responses ranging from “*not at all true*” to “*very true*,” and a higher total score reflected greater intrinsic motivation for a specified task.

Treatment satisfaction was assessed using the Client Satisfaction Questionnaire (CSQ) (Tachimori and Ito, 1999), which is an 8-item 4-case Likert scale that measures satisfaction with treatment and care, with a higher score indicating a higher treatment satisfaction.

The PANSS was measured by a psychiatrist while the BACS, BCIS, IMI, and CSQ measurements were performed by an occupational therapist.

### Statistical Analyses

A per protocol analysis was performed. The target population of the analysis consisted of all patients with schizophrenia and schizoaffective disorder who met all inclusion criteria and no exclusion criteria. The *t*-test and or χ^2^-test were used for inter-group comparisons of basic demographic information. For the analysis of assessment scores (i.e., scores on the evaluation scale), the two-way repeated-measures ANOVA was used, with timing (baseline versus post-intervention) and group (OT + MCT versus OT-only) as the within-subjects factor and between-subjects factor. The chi-square test was used to compare the number of people who were readmitted within one year of discharge. The software used for statistical analysis was the Bell Curve for Excel (version 3.21) with a significance level of 5%.

## Results

### Demographic Information of the Participants

**FIGURE 1:**
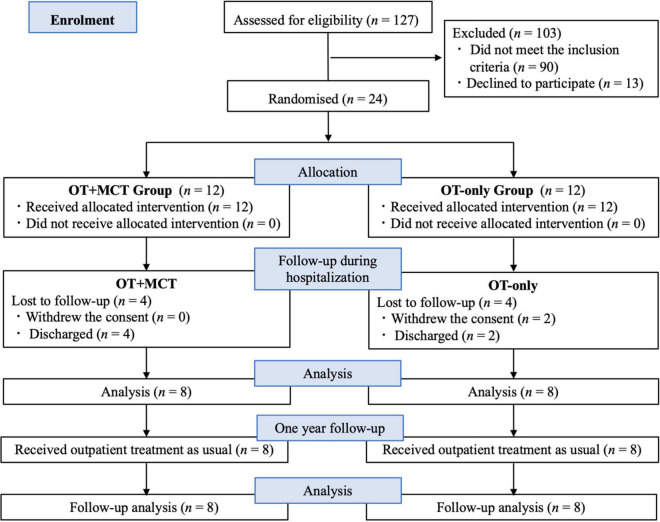
Flow chart of this study.

A flowchart of the study is shown in Figure 1. Eligibility was evaluated in 127 patients who were admitted to the emergency psychiatric ward during the study period. Of these, 90 patients did not meet the inclusion criteria, and 13 patients refused to participate in the study, leaving 24 participants. The 90 patients who did not meet the criteria included 13 patients younger than 20 years or older than 66 years, 23 patients with comorbidities, 35 patients with unstable conditions, and 19 short-term inpatients (e.g., less than one week). Of the 24 patients, 12 were randomly allocated to the OT + MCT and OT-only groups. During the follow-up period, four patients in the OT + MCT group were discharged from the hospital. In the OT-only group, two patients withdrew informed consent, and two patients were discharged from the hospital. Therefore, eight patients were included in each group in the analyses.

The patients’ demographic characteristics are shown in Table 1. The mean (±SD) ages of the patients showed no significant difference between the two groups, being 44.25 ± 8.58 years in the OT + MCT group and 43.25 ± 7.98 years in the OT-only group. In addition, inter-group comparisons showed no significant difference in sex, number of people living in the same residence, years of education, disease duration, number of hospitalizations, time from hospitalization to OT initiation, OT implementation duration, or duration of hospitalization. The antipsychotic dose (chlorpromazine equivalent) tended to be higher in the OT + MCT group, but the difference was not significant. All 16 patients included in the analyses were diagnosed with schizophrenia.

**TABLE 1:** Demographic characteristics of the participants.

Variable	Total (*n* = 16)		OT + MCT (*n* = 8)		OT-only (*n* = 8)		*p*
	Mean	(SD)	Mean	(SD)	Mean	(SD)	
Age (years)	43.75	(8.55)	44.25	(8.54)	43.25	(7.98)	0.82
Sex (male/female), *n*	8	8	5	3	3	5	0.32
Cohabitant (*n*)	1.00	(0.73)	1.00	(0.71)	1.63	(0.70)	0.12
Education (years)	14.50	(2.58)	13.75	(1.85)	15.25	(2.81)	0.26
Disease duration (years)	19.81	(12.37)	21.75	(12.49)	17.88	(11.11)	0.55
Number of hospital stays (times)	3.00	(2.45)	3.63	(3.12)	2.38	(0.86)	0.32
Starting OT from hospitalization (days)	27.75	11.30	28.50	(13.31)	27.00	(7.83)	0.80
OT implementation time (hours)	35.88	(15.64)	36.44	(13.51)	39.06	(17.46)	0.74
Length of hospital stays (days)	78.63	(28.11)	81.50	(28.60)	75.75	(29.26)	0.70
Antipsychotic (mg/day)^a^	510.30	(356.52)	670.60	(313.53)	350.00	(297.71)	0.07

*^a^Chlorpromazine equivalent dose.*

### Changes in the Assessment Scores

Changes in the assessment scores in the OT + MCT and OT-only groups were analyzed using the two-way repeated-measures ANOVA (Table 2).

**TABLE 2:** Assessment score analysis by two-way repeated-measures ANOVA.

Measure	Time	OT + MCT (*n* = 8)		OT-only (*n* = 8)		*F*			*ES* (η*^2^*)
		Mean	(SD)	Mean	(SD)	Time	Group	Time × Group	
**BACS**									
⋅ Verbal memory	Baseline	−1.46	(1.01)	−0.77	(1.08)	0.39	1.20	0.58	0.01
	Post	−1.20	(0.91)	−0.80	(0.98)				
⋅ Working memory	Baseline	−0.48	(1.02)	−0.75	(0.64)	0.02	0.00	1.11	0.02
	Post	−0.75	(1.20)	−0.54	(1.07)				
⋅ Motor speed	Baseline	−2.03	(1.34)	−2.12	(0.90)	1.29	0.14	0.91	0.02
	Post	−1.98	(0.72)	−1.56	(0.76)				
⋅ Verbal fluency	Baseline	−0.83	(1.14)	−0.58	(0.67)	5.16*	0.09	1.05	0.01
	Post	−0.46	(0.83)	−0.44	(0.80)				
⋅ Attention	Baseline	−1.24	(1.08)	−1.38	(0.87)	2.07	0.02	0.16	0.00
	Post	−1.10	(1.32)	−1.12	(0.82)				
⋅ Executive function	Baseline	−1.45	(1.46)	−0.77	(2.34)	6.37*	0.09	2.30	0.02
	Post	−0.32	(1.02)	−0.49	(1.56)				
⋅ Composite score	Baseline	−1.25	(0.94)	−1.06	(0.88)	7.68*	0.14	0.10	0.00
	Post	−0.95	(0.74)	−0.82	(0.65)				
**PANSS**									
⋅ Positive	Baseline	21.88	(5.53)	21.50	(8.00)	10.03**	0.33	0.80	0.02
	Post	13.63	(3.24)	16.88	(6.35)				
⋅ Negative	Baseline	20.13	(9.99)	18.50	(4.27)	19.00**	0.00	2.58	0.02
	Post	13.63	(5.94)	15.50	(5.20)				
⋅ General psychopathology	Baseline	44.13	(9.88)	36.38	(7.92)	20.29**	0.51	6.04*	0.08
	Post	29.25	(6.50)	32.00	(5.79)				
⋅ Total score	Baseline	86.13	(21.38)	78.50	(13.36)	37.88**	0.00	4.35	0.04
	Post	55.00	(9.55)	63.13	(10.35)				
**BCIS**									
⋅ Self-reflectiveness	Baseline	12.38	(4.06)	9.88	(4.31)	1.70	5.55*	0.32	0.01
	Post	15.88	(3.92)	11.25	(5.54)				
⋅ Self-certainty	Baseline	6.88	(4.88)	6.63	(4.24)	7.18*	0.04	0.09	0.00
	Post	5.38	(4.06)	4.75	(2.90)				
⋅ Composite index	Baseline	5.63	(6.06)	3.50	(8.22)	4.82*	1.15	0.27	0.01
	Post	10.50	(5.89)	6.50	(4.53)				
**IMI**									
⋅ Interest/enjoyment	Baseline	33.38	(9.11)	30.88	(10.10)	1.05	0.26	0.01	0.00
	Post	34.50	(7.55)	32.25	(9.38)				
⋅ Perceived choice	Baseline	34.50	(10.16)	28.63	(11.28)	1.00	1.33	0.06	0.00
	Post	35.63	(6.73)	30.50	(8.63)				
⋅ Value/usefulness	Baseline	38.50	(9.55)	31.88	(9.08)	0.72	1.73	0.20	0.00
	Post	39.00	(8.60)	33.50	(8.43)				
⋅ IMI total	Baseline	106.38	(19.31)	91.38	(30.22)	2.84	1.30	0.22	0.00
	Post	109.13	(17.82)	96.25	(23.82)				
CSQ	Post	26.13	(3.06)	24.00	(2.00)	*t* = 1.54, *p* = 0.15, *Cohen’s* d = 0.82			

*Two-way repeated measures analysis of variance.*

*Time (within-subjects factor), Group (between-subjects factor), Time × Group (interaction)*

*ES, Effect size (η^2^), interaction of Time × Group; η^2^ = 0.01 (small), η^2^ = 0.06 (medium), η^2^ = 0.14 (large)*

*BACS, Brief Assessment of Cognition in Schizophrenia; PANAS, Positive and Negative Syndrome Scale; BCIS, Beck Cognitive Insight Scale; IMI, Intrinsic Motivation Inventory; CSQ, Client Satisfaction Questionnaire.*

*p < 0.05.

**p < 0.01.

Each of the BACS scores showed a tendency toward an increase in the post-intervention evaluation. Verbal fluency showed a significant difference in the within-subjects factor (F [1, 14] = 5.16, *p* = 0.04, η^2^ = 0.02); however, between-subjects factor (F [1, 14] = 0.09, *p* = 0.77, η^2^ = 0.01) and interactions (F [1, 14] = 1.05, *p* = 0.32, η^2^ = 0.01) were not significantly different. The executive function showed a significant difference in the within-subjects factor (F [1, 14] = 6.37, *p* = 0.02, η^2^ = 0.04), but no significant difference in the between-subjects factor (F [1, 14] = 0.09, *p* = 0.77, η^2^ = 0.01) and interactions (F [1, 14] = 2.29, *p* = 0.15, η^2^ = 0.02). The composite score showed a significant difference in the within-subjects factor (F [1, 14] = 7.68, *p* = 0.02, η^2^ = 0.03), but no significant difference in the between-subjects factor (F [1, 14] = 0.14, *p* = 0.71, η^2^ = 0.01) and interactions (F [1, 14] = 0.10, *p* = 0.76, η^2^ = 0.00). There were no significant differences in verbal memory, working memory, motor speed, or attention for the within-subjects factor, between-subjects factor, and interactions.

With respect to PANSS, there was a tendency for the score to decrease in the post-intervention evaluation. Positive symptoms were significantly different for the within-subjects factor (F [1, 14] = 10.03, *p* = 0.007, η^2^ = 0.22), but not for the between-subjects factor (F [1, 14] = 0.33, *p* = 0.57, η^2^ = 0.01) and interactions (F [1, 14] = 0.79, *p* = 0.39, η^2^ = 0.02). Negative symptoms showed a significant difference in the within-subjects factor (F [1, 14] = 19.00, *p* = 0.00, η^2^ = 0.11), but between-subjects factor (F [1, 14] = 0.00, *p* = 0.97, η^2^ = 0.00) and interactions (F [1, 14] = 2.58, *p* = 0.13, η^2^ = 0.02) were not significantly different. General psychopathology showed a significant difference in the within-subjects factor (F [1, 14] = 20.29, *p* = 0.00, η^2^ = 0.24) and interactions (F [1, 14] = 6.04, *p* = 0.03, η^2^ = 0.08). However, no significant difference was found for the between-subjects factor (F [1, 14] = 0.51, *p* = 0.49, η^2^ = 0.02). The PANSS total score showed a significant difference in the within-subjects factor (F [1, 14] = 37.88, *p* = 0.00, η^2^ = 0.38), but no significant difference in the between-subjects factor (F [1, 14] = 0.00, *p* = 0.97, η^2^ = 0.00) and interactions (F [1, 14] = 4.35, *p* = 0.06, η^2^ = 0.04).

The mean scores of the General Psychopathology subscale items of the PANSS are shown in Table 3. In the post evaluation of the OT + MCT group, the average scores of anxiety, tension, motor retardation, uncooperativeness, poor attention, poor impulse control, preoccupation, and active social avoidance decreased by 1.00 or more. On the other hand, there was no item whose average score decreased by 1.00 or more in the post-evaluation in the OT-alone group.

**TABLE 3:** Changes in the mean of the general psychopathology scale of PANSS.

		OT + MCT (*n* = 8)			OT-alone (*n* = 8)		
	Items	Baseline	Post	Difference	Baseline	Post	Difference
1	Somatic concern	2.00	1.63	−0.38	2.38	2.00	−0.38
2	Anxiety	3.38	2.13	−1.25	3.00	2.38	−0.63
3	Guilt feelings	2.38	1.75	−0.63	1.00	1.00	0.00
4	Tension	2.88	1.88	−1.00	1.88	1.63	−0.25
5	Mannerisms and posturing	3.13	2.25	−0.88	2.00	2.00	0.00
6	Depression	2.25	2.00	−0.25	2.50	2.63	0.13
7	Motor retardation	3.00	2.00	−1.00	2.13	2.25	0.13
8	Uncooperativeness	3.00	1.63	−1.38	2.25	2.00	−0.25
9	Unusual thought content	3.88	3.00	−0.88	3.25	3.00	−0.25
10	Disorientation	1.50	1.00	−0.50	1.38	1.25	−0.13
11	Poor attention	2.75	1.50	−1.25	2.25	2.00	−0.25
12	Lack of judgment and insight	3.50	2.75	−0.75	2.50	2.50	0.00
13	Disturbance of volition	2.38	1.63	−0.75	2.38	1.75	−0.63
14	Poor impulse control	2.50	1.50	−1.00	2.13	2.00	−0.13
15	Preoccupation	2.75	1.50	−1.25	1.75	1.63	−0.13
16	Active social avoidance	3.00	1.63	−1.38	2.25	2.13	−0.13

With respect to BCIS, self-reflectiveness was significantly different for the between-subjects factor (F [1, 14] = 5.55, *p* = 0.03, η^2^ = 0.08); however, the within-subjects factor (F [1, 14] = 1.70, *p* = 0.21, η^2^ = 0.01) and interactions (F [1, 14] = 0.32, *p* = 0.58, η^2^ = 0.00) were not significantly different. Self-certainty was significantly different for the within-subjects factor (F [1, 14] = 7.18, *p* = 0.02, η^2^ = 0.04), but between-subjects factor (F [1, 14] = 0.04, *p* = 0.84, η^2^ = 0.00) and interactions (F [1, 14] = 0.09, *p* = 0.77, η^2^ = 0.00) were not significantly different. The BCIS composite index showed a significant difference in the within-subjects factor (F [1, 14] = 4.82, *p* = 0.04, η^2^ = 0.08), but between-subjects factor (F [1, 14] = 1.15, *p* = 0.30, η^2^ = 0.05) and interactions (F [1, 14] = 0.27, *p* = 0.61, η^2^ = 0.01) were not significantly different.

Regarding IMI, there were no significant differences in the within-subjects factor, between-subjects factor, and interactions in Interest/enjoyment, Perceived choice, Value/usefulness, and IMI total. In addition, the inter-group comparison of the CSQ as a post-intervention evaluation showed no significant difference.

### Readmission Within 1 Year

Both the OT + MCT group and the OT alone group received outpatient treatment after discharge. The usual treatment included physician consultation and medication. The number of patients who were readmitted within 1 year following discharge was 2 (25%) in the OT + MCT group and 6 (75%) in the OT alone group; the readmission in the OT + MCT group was significantly lower (*p* = 0.046).

## Discussion

### Applicability of Metacognitive Training in an Emergency Psychiatric Ward

Previous studies have reported the efficacy of MCT (Moritz et al., 2011, 2013, 2014a, 2014b; Hosono et al., 2013; Morimoto et al., 2015; Ishikawa et al., 2020). The patients were either hospitalized in general psychiatric wards or were attending the hospital on a day-care basis, and there have thus been no reports of patients hospitalized in emergency psychiatric wards. The present study was the first to investigate the efficacy of MCT in patients with schizophrenia in a Japanese emergency psychiatric ward. However, the mean (±SD) disease duration was 21.75 ± 12.49 years in the OT + MCT group and 17.87 ± 11.10 years in the OT-only group, and the mean (±SD) number of admissions to the hospital was 3.63 ± 3.12 in the OT + MCT group and 2.38 ± 0.86 in the OT-only group. Therefore, the study included numerous patients who had been re-hospitalized due to recurrence of schizophrenia or recrudescence of psychiatric symptoms. There was about a month between the admission and the start of OT. The patient’s acute psychiatric symptoms were relieved to some extent by the inpatient treatment during this period, and consequently, the baseline evaluation showed moderate cognitive impairment and mild to moderate psychiatric symptoms. Therefore, although the participants in this study were admitted to a psychiatric emergency ward, they were considered to be patients in the recovery phase who had already passed the acute and subacute phases.

According to a systematic review by Jiang et al. (2015), the MCT dropout rate of schizophrenia patients is less than 30%, which is approximately the same as the dropout rate in the control group. In comparison, at 33% in both the OT + MCT and OT-only groups, the dropout rate in the present study was rather high. Of the eight patients who dropped out of this study before completion, six were discharged from the hospital(75%), and two withdrew consent (25%). It is possible that the dropout rate was influenced by the healthcare system, with the patients expected to be discharged within 3 months. However, in the MCT conducted in a small group, the burden on the individual participants was dispersed; the interactive and game-like fun (Moritz et al., 2010) made it possible to apply the MCT to patients in the psychiatric emergency ward. In addition, if patients require individualized support due to delusional symptoms, it is possible to implement individualized MCT (MCT+) (Moritz et al., 2010), although this was not used in this study.

### Effectiveness of Metacognitive Training

The results of the two-way repeated-measures ANOVA showed that the score changes of BACS, PANSS, and BCIS appeared to differ greatly depending on the within-subjects factor (before and after).

Brief Assessment of Cognition in Schizophrenia z-scores increased after intervention in all domains, but there were no significant differences in between-subjects factors and no benefit of MCT on neurocognitive function. Baseline-assessed BACS-z scores were notably lower for motor speed and attention in both groups, and verbal memory and executive function were also moderately or severely impaired in the OT + MCT group. These moderate or greater deficits in attention, memory, and executive function may have made it more difficult for patients to engage in MCT and limited the effectiveness of the training. For post-acute schizophrenic patients with impaired attention and memory, the use of MCT-Acute,^2^ which reduces module complexity and time, may need to be considered to avoid psychological upset and confusion.

Concerning PANSS, interactions between the within-subjects factor and the between-subjects factor were found for general psychopathology, and the OT + MCT group tended to show more alleviation of psychiatric symptoms than the OT-only group. At baseline assessment, the OT + MCT group took more antipsychotics than the OT alone group (*p* = 0.07). In the OT + MCT group, the appropriate dose of antipsychotic medication calmed the psychotic symptoms, and the MCT session facilitated interaction among the participants, and the experience of enjoying the learning task may have further accelerated the improvement of psychotic symptoms. As shown in Table 3, in the OT + MCT group, the mean scores of anxiety, tension, motor retardation, uncooperativeness, poor attention, poor impulse control, preoccupation, and active social avoidance decreased by more than 1.00 points. These improvements could be attributed to the psychosocial therapeutic effects of MCT conducted in a group setting, such as paying attention to the tasks presented, listening to what the staff and participants had to say, thinking about the answers, and expressing one’s ideas, which may have facilitated the alleviation of psychiatric symptoms and reduced the risk of readmission.

The effects of MCT in alleviating psychiatric symptoms, especially delusions and other positive symptoms, have been previously reported (Moritz and Woodward, 2007; Kumar et al., 2010; Moritz et al., 2011, 2014a; Briki et al., 2014; Pankowski et al., 2016), and MCT has been shown to increase cognitive insight (Lam et al., 2015). The two-way repeated measures ANOVA in this study showed a significant difference in the between-subjects factor in BCIS self-reflectiveness (*p* = 0.03). Thus, in the OT + MCT group, the implementation of MCT may have contributed to the improvement in patients’ self-reflectiveness. There have been reports of a single-route model, in which interventions targeting visual perception, cognition, etc., have resulted in beneficial effects in subsequent processing stages and on the functional outcome (Green et al., 2012). During MCT sessions, numerous animations, pictures, etc. are presented in the PowerPoint as training materials to improve/reduce areas such as causal attribution, jumping to conclusions, confirmation bias, theory of mind, and verbal memory errors. These modules activated the patients’ visual cognition during the MCT sessions and also promoted active thinking related to prediction and inference, which may have contributed to their improved self- reflectiveness.

The importance of motivation in affecting the outcome of the cognitive-improvement approach to treat schizophrenia has been pointed out (Velligan et al., 2006; Green et al., 2012). In the present study, IMI, which reflects intrinsic motivation, showed only minor changes in scores in the OT + MCT and OT-only groups; and no significant improvement was found. In addition, CSQ, which represents satisfaction with treatment, showed no significant differences between the groups. These findings may be linked to the short intervention period. It is necessary to continue investigating the contribution of MCT to intrinsic motivation and satisfaction with treatment.

Finally, it is noteworthy that the OT + MCT group had significantly fewer readmissions within 1 year of discharge. However, details of post-discharge care and living conditions in both groups were not investigated. Considering the sample size, the preventive effect of MCT on readmission (recurrence) is not understood; therefore, this should be taken into consideration.

### Limitations and Future Challenges

There are several limitations to this study.

First this study had a small sample size and was an open-label trial. Tendencies toward alleviation/improvement of psychiatric symptoms and cognitive insight were found in the OT + MCT group; however, the reliability of these results is limited due to the small sample size. The study was positioned as preliminary; therefore, it is essential to reassess the findings in additional studies with larger sample sizes. In addition, the effects of MCT on neurocognitive function, intrinsic motivation, and satisfaction with treatment are unclear, and further research is needed.

Second, only 24 patients (19%) were enrolled, even though 127 participants were assessed for eligibility. Therefore, it is possible that the 24 enrolled patients do not represent the clinical characteristics of patients admitted to the emergency psychiatric ward. It is also clinically important to investigate intervention strategies for patients with unstable conditions who were not enrolled in this study.

Third, the MCT effects in this study were short-term effects during the hospitalization period; and it is necessary to perform further studies to assess the long-term effects on the functional prognosis, especially on the adequacy of patients’ social life. To clarify the preventive effect of MCT on readmission, it is necessary to increase the sample size in the follow-up and investigate the mental healthcare and individual living conditions received by the patients in detail.

In conclusion, in both the OT + MCT and OT-only groups, the discontinuation rate of the patients was 33% due to hospital discharge in 75% of cases; this shows that early discharge from emergency psychiatric wards readily influences MCT discontinuation. The two-way repeated-measures ANOVA showed interactions between the timing and group in general psychopathology in PANSS, and the alleviation of symptoms was more marked in the OT + MCT group. In the OT + MCT group, tendencies toward alleviation/improvement of psychiatric symptoms and self-reflectiveness were found, suggesting that MCT is effective for patients with schizophrenia in emergency psychiatric wards. To determine whether MCT during hospitalization contributes to the prevention of readmission, it is necessary to increase the sample size and to examine mental health care and individual life background in detail in follow-up surveys.

## Data Availability Statement

The datasets presented in this study can be found in online repositories. The names of the repository/repositories and accession number(s) can be found in the article/Supplementary Material.

## Author Contributions

SH and MK planned and designed this study and created the manuscript, figure, and tables. SH, AT, KK, and KE contributed to the acquisition and analysis of the data. All authors contributed to the article and approved the submitted version.

## Conflict of Interest

The authors declare that the research was conducted in the absence of any commercial or financial relationships that could be construed as a potential conflict of interest.

## Publisher’s Note

All claims expressed in this article are solely those of the authors and do not necessarily represent those of their affiliated organizations, or those of the publisher, the editors and the reviewers. Any product that may be evaluated in this article, or claim that may be made by its manufacturer, is not guaranteed or endorsed by the publisher.

## Footnotes

1
www.uke.de/mkt

2
www.uke.de/mct-acute
